# Hungarian male water polo players’ body composition can predict specific playing positions and highlight different nutritional needs for optimal sports performance

**DOI:** 10.1186/s13102-022-00560-9

**Published:** 2022-09-05

**Authors:** Péter Fritz, Réka Fritz, Lívia Mayer, Boglárka Németh, Judit Ressinka, Pongrác Ács, Csilla Oláh

**Affiliations:** 1 Faculty of Health Science, University of Miskolc, Miskolc-Egyetemváros, Building Stefánia, Miskolc, 3515 Hungary; 2 Doctoral School of Clinical Medicine, University of Szeged, Szeged, Hungary; 3 Superfoods Ltd, Budapest, Hungary; 4 FIT360 Fitness Studio, Budapest, Hungary; 5 Institute of Physiotherapy and Sport Science, University of Pécs, Pecs, Hungary; 6 Department of Urology, University of Duisburg-Essen, Essen, Germany

**Keywords:** Water polo, Body composition, Anthropometrics, Playing position, Dietary habits, Sport nutrition, Laboratory parameters

## Abstract

**Background:**

Water polo is unique among aquatic—and generally other—sports as it includes cyclic elements typical in swimming and acyclic elements occurring mainly in ball games. Moreover, water polo demands high level of technical and tactical skills. Players need an optimal nutritional and physical condition to achieve high athletic performance, which is to a great extend influenced by nutritional habits. We aim to highlight possible shortfalls in players’ nutritional intake in relation to positions played within the team.

**Methods:**

In the present study, we determined the anthropometric and body composition characteristics, dietary habits and laboratory parameters of elite adult male water polo players (n = 19) before the start of the championship and at the end of the regular season, which meant a 4-month intervention period. Analyses of body composition characteristics and nutritional habits were performed using bioimpedance analyzer InBody 770 and a 3-day nutrition diary, respectively. Paired-sample t-test were used to determine the differences between the variables measured before and after the championship. Correlations between the anthropometric and body composition characteristics and different serum parameters were analyzed using linear correlation calculation. K-mean cluster analysis was performed using the anthropometric and body composition characteristics of the athletes.

**Results:**

Based on anthropometric and body composition characteristics, players can be divided into two significantly different clusters that shows an association with specific playing positions. Cluster I included goalkeepers and wing players, while defenders, centers, and shooters belonged to Cluster II. We observed significant differences in the physical composition and slight but not significant differences in nutritional habits of the clusters. Cluster I players were 5 cm shorter on average, while their mean body weight, skeletal muscle mass and body fat mass data were lower by 19 kg, 7 kg, and 7 kg, respectively. We studied the correlation between initial anthropometric and body composition parameters and the changes in laboratory parameters before and after the regular season. As a result, we detected numerous significant differences between the two clusters, such as the changes in glucose and magnesium levels, which showed a strong correlation with several body composition parameters in cluster II, but did not in cluster I.

**Conclusions:**

Cluster differences between anthropometric and body compositional characteristics, and the changes in laboratory parameters can help to develop position-specific training and nutritional recommendations in the future. Therefore, the results may be applicable in sport sciences for elite athletes and sports coaches.

**Supplementary Information:**

The online version contains supplementary material available at 10.1186/s13102-022-00560-9.

## Background

Nutritional habits significantly influence the adequate sports performance of athletes. Therefore, the number of scientific publications in the field of sports nutrition has multiplied over the past ten years. International recommendations have already become available to determine the optimal daily intake of protein, carbohydrate, and fat in relation to exercise intensity [1–6]. However, it is important to note that in addition to sports-specific nutrition, position-specific recommendations are also needed according to the body composition characteristics and energy requirements of the players. To the best of our knowledge, no position-specific nutritional recommendations have been described for water polo players yet. Although, generally fewer studies have been published in the field of water polo compared to other sport fields, such as swimming. However, position-specific data of anthropometric and body composition characteristics are available [7, 8].

In a previous study, anthropometric characteristics, physiology, and conditional status of junior-aged water polo players were measured. The authors found position-specific differences in the anthropometric characteristics of the players, which probably also affected the results of their swimming tests [7]. Thus, these studies highlighted the relevance of body compositional measurements for water polo players. In another study, reference values ​​of markers for measuring anthropometric and body composition characteristics for players of 21 sports were developed [9]. Unfortunately, water polo was not among the 21 examined sports. Professional sports with a high number of training sessions and games require players to be in excellent physical, mental and nutritional condition, as high volumes can lead to physical and mental fatigue and loss of performance. Furthermore, the high stakes of the match can put an extra burden on the players as well [10]. Sufficient fluid intake is also relevant to adequate sports performance [11]. These studies can provide a good basis for developing position-specific training programs supported by nutritional recommendations and taking the anthropometric and body composition characteristics of players into account. Nutritional status influences body composition and recovery time after training sessions or games, thereby examining markers (such as amino acids, urea, glucose, free fatty acids, various vitamins, etc.) that provide detailed information about nutritional status is essential for optimizing sports performance. [3, 12]. However, nutritional deficiencies occur commonly in athletes, therefore frequent monitoring of the above-mentioned markers can identify deficiencies, especially when higher nutritional demands increase due to the higher training volume [12]. However, we have also taken into consideration that the examination of a single marker does not provide sufficient information and each marker has a reference range.

In the present study, we analyzed the correlation between anthropometric and body composition characteristics, dietary habits, and laboratory parameters of elite adult male athletes playing in a world-class Hungarian water polo team, in the Gymnastic Club of Ferencváros (Ferencvárosi Torna Club, (FTC), in order to determine possible differences between players with different playing-positions, especially taking account of players’ nutritional intake.

## Methods

### Participants

The present study involved 19 adult athletes from an elite Hungarian male water polo team (FTC). All participants had sufficient years of competitive water polo experience (15.2 ± 3.4 years). The study included 4 goalkeepers, 7 wings, 3 defenders, 3 centers, and 2 shooters with an average age of 25.2 ± 1.22 years. The study was performed in accordance with the Declaration of Helsinki and the Regional Local Committee of Science and Research Ethics approved the study protocol (BORS/4-1/2020). All players received the detailed description of the experiment before providing their written informed consent.

### Design and data collection

The study was performed between August and December of 2020 in Budapest. The measured variables, such as anthropometric and body composition characteristics, nutritional habits, and laboratory parameters were analyzed in two different sampling times, before the start of the championships and at the end of the regular season, which covered a 4-month intervention period. During the research period, the strength endurance training was replaced with more tactical training and games. The changes of variables before and after the intervention period were also examined.

To assess socio-demographic data, health status and sports habits of the players, a self-administrated questionnaire was compiled. The life quality of players was measured using the validated SF36 questionnaire [13, 14]. The participants’ energy, and their macro- and micronutrient intake were analyzed based on a 3-day nutrition diary. Extensive body composition analysis was measured using InBody 770 (InBody Co., Ltd., South Korea), which is based on bioelectrical impedance analysis and can establish a person’s body composition with a > 92% accuracy compared to DEXA Scan [15]. During the analysis, the following parameters were determined: weight (kg), body mass (kg), skeletal muscle mass (kg), body fat mass (kg), fat-free mass (kg), total body water (l), extracellular water (l), intracellular water (l), protein mass (kg), mineral mass (kg), basal metabolic rate (kcal), body cell mass (kg) and overall fitness score (InBody Score). Blood samples were collected before the first training on an empty stomach. After centrifugation (2.500 rpm for 10 min) the following parameters were analyzed: glutamate–oxaloacetate-transaminase (GOT), glutamate-pyruvate-transaminase (GPT), gamma-glutamyl transferase (gamma GT), serum creatinine, serum sodium, serum potassium, serum chloride, serum calcium, serum magnesium, cholesterol, HDL- cholesterol, LDL- cholesterol, glucose, triglyceride, urea, serum creatinine kinase, serum uric acid, serum ferritin, Vitamin B12, Vitamin D and routine blood parameters such as red blood cells (RBC), white blood cells and hemoglobin. The measurements were performed in the pre- and post-intervention periods, in August and December.

### Statistical analysis

Paired-sample t-test were used to determine the differences between the variables measured in the pre-and post-intervention periods. Correlations between the anthropometric and body composition characteristics and different serum parameters, were performed using linear correlation calculation. In the cluster analysis, based on the anthropometric and body composition characteristic data, *k*-means were used, and Euclidean distances were calculated. Statistical analyses were performed using the SPSS statistical software (IBM SPSS Statistics for Windows, version 26, IBM Corp., Armonk, NY, USA).

## Results

The socio-demographic data show that each participant involved in the study habitually lived in Budapest during the 4-month intervention period. However, 10% indicated another large city as their permanent residence and further 30% named some other small towns in Hungary. The permanent residence could influence the current eating habits. The players were between 18 and 34 years of age. The average body weight and height were 93.7 kg (± 2.66) and 193.5 cm (± 1.32), respectively.

At the pre- and post-intervention measurements, the players completed a validated life quality questionnaire (SF36) to assess their mental condition and overall health status. Due to the inconsistent views in the literature for evaluation of interval scales, the results of the life quality questionnaire were compared with both parametric and non-parametric statistical tests. Although the degree of significance levels differs in several cases, the results show a similar trend in both parametric and non-parametric statistical tests (Additional file 1: Table S1). Of the 36 concepts, only 3 showed no significant differences between the two sampling times. According to the test results, unfavorable significant changes were detected in the mental and perceived physical state of athletes, in almost all cases. In the post-intervention period, the players felt generally more tired and reported unfavorable changes in their health status.

**Fig. 1:**
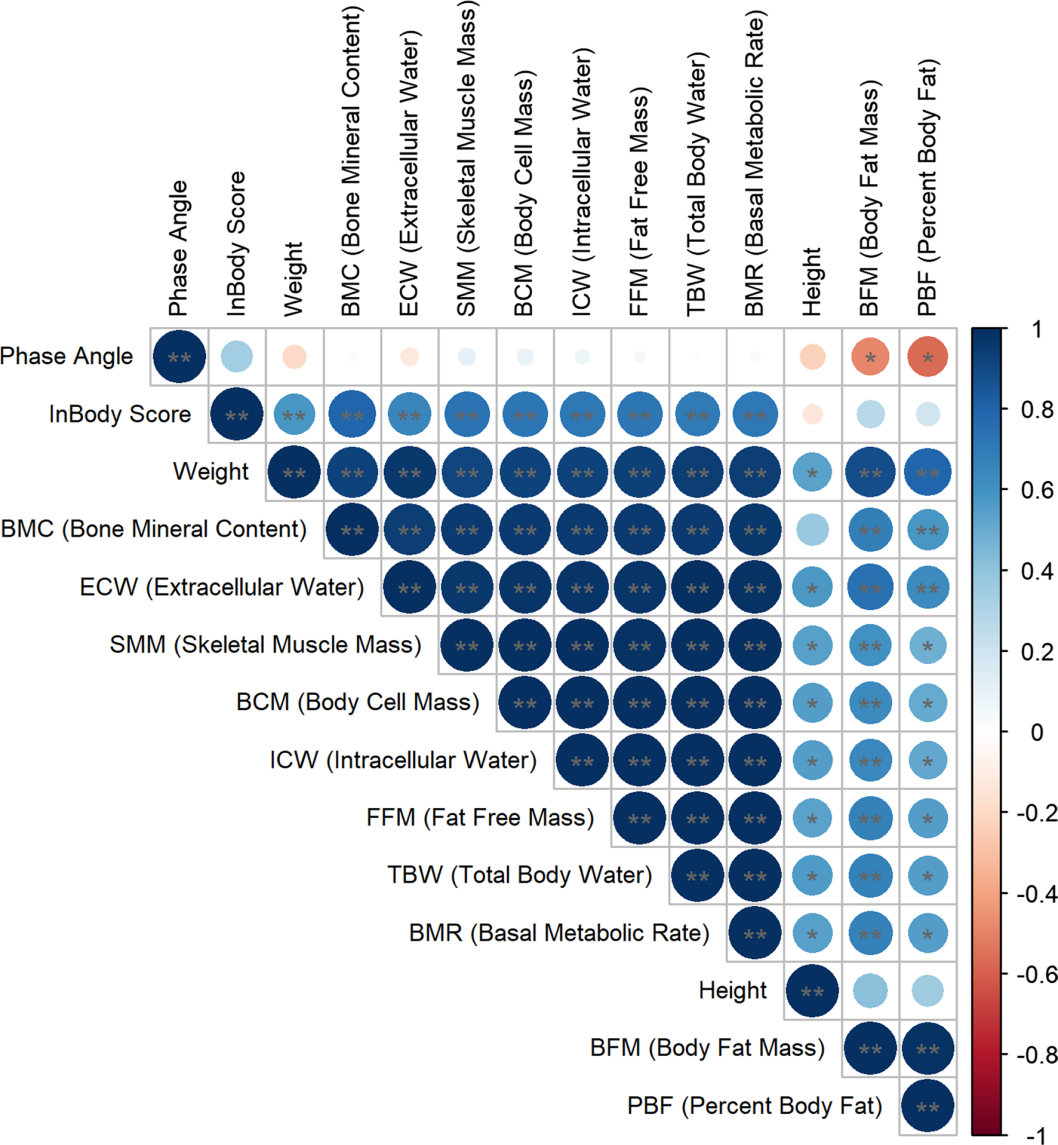
Correlation between the main anthropometric and body composition characteristics. Dark blue dots represent a stronger positive correlation between the variables according to the scale (r > 0.8), while red dots represent a negative correlation. Statistical significance was designated by **p ≤ 0.01, *p ≤ 0.05

The correlation analysis between the anthropometric data and body composition characteristics showed a strong positive correlation in most parameters (Fig. 1). The InBody Score strongly correlated with numerous parameters except body height, body fat mass, percent body fat, and phase angle.

Based on the anthropometric and body composition characteristics, we could classify the athletes into two distinct, significantly different clusters. Except for the value of the phase angle and body height, we found significant differences in all cases between the two clusters. Athletes classified into Cluster II were taller and hadstronger physique and more intense metabolism (Table 1). It is important to note that we found an association between the clusters and the specific positions of the players. Cluster I included 4 goalkeepers and 7 wingers, while Cluster II included 3 defenders, 3 centers, and 2 shooters.

**Table 1:** Anthropometric and body composition characteristics of the clusters

Body composition characteristics	Cluster I (n = 11)	Cluster II (n = 8)	F-test value
Body height (cm)	191.090 ± 4.056	196.875 ± 4.559	5.88
Body mass (kg)	85.672 ± 9.002	104.838 ± 10.953	40.20**
Skeletal muscle mass (kg)	44.355 ± 3.572	51.250 ± 2.645	31.21**
Body fat mass (kg)	8.682 ± 3.875	15.575 ± 7.314	14.58*
Percent body fat (%)	10.073 ± 3.277	14.650 ± 5.545	9.35*
Fat-free mass (kg)	77.018 ± 6.196	89.738 ± 4.632	38.33**
Total body water (l)	56.400 ± 4.413	65.175 ± 3.323	38.08**
Body cell mass (kg)	50.700 ± 3.990	58.488 ± 2.880	36.17**
Extracellular water ratio	21.245 ± 1.660	24.325 ± 1.365	37.53**
Intracellular water ratio	35.400 ± 2.778	40.930 ± 2.031	36.68**
Whole body phase angle	6.682 ± 0.456	6.713 ± 0.506	0.02
Basal metabolic rate (kcal)	2033.091 ± 133.767	2297.750 ± 100.183	38.37**
Minerals (kg)	5.235 ± 0.544	6.419 ± 0.445	22.69**
InBody Score	87.636 ± 6.346	94.750 ± 4.086	6.43*

Statistical significance between clusters was designated by **p ≤ 0.01, *p ≤ 0.05

Then, we also examined changes that occurred in the anthropometric and body composition characteristics during the intervention period. We detected significant differences between mineral compositions, which decreased significantly by the end of the 4-month intervention period (Table 2).

**Table 2:** Changes in the anthropometric and body composition characteristics before and after the intervention period

Variables	Before the start of the championship	After the championship	T-test value
*Body composition characteristics*			
Body height (cm)	193.526 ± 5.787	193.474 ± 5.591	–
Body mass (kg)	93.742 ± 11.596	94.226 ± 11.859	0.127
Skeletal muscle mass (kg)	47.258 ± 4.347	46.884 ± 4.155	− 0.271
Body fat mass (kg)	11.584 ± 5.146	12.289 ± 5.894	0.392
Percent body fat (%)	12.000 ± 3.897	12.642 ± 4.451	0.473
Fat-free mass (kg)	82.374 ± 7.752	81.805 ± 7.120	− 0.235
Total body water (l)	60.095 ± 5.353	59.826 ± 5.117	− 0.158
Body cell mass (kg)	53.979 ± 4.790	53.742 ± 4.567	− 0.156
Extracellular water ratio	22.400 ± 2.033	22.300 ± 1.967	− 0.154
Intracellular water ratio	37.721 ± 3.838	37.526 ± 3.182	− 0.183
Whole body phase angle	6.695 ± 0.468	6.737 ± 0.466	0.277
Basal metabolic rate (kcal)	2144.526 ± 161.399	2136.842 ± 153.793	− 0.150
Minerals (kg)	5.733 ± 0.794	4.783 ± 0.580	− 4.211**
InBody Score	90.632 ± 6.890	90.053 ± 5.421	− 0.287

Statistical significance was designated by **p ≤ 0.01, *p ≤ 0.05

We examined the dietary habits of the athletes using a 3-day nutrition diary. During the quantitative measurements, the intake of macronutrients like protein, carbohydrate, fat, also fiber and energy was determined, then these data were normalized to body weight. Nutrition habits were analyzed separately in the two clusters determined earlier by anthropometric and body composition characteristics. We found that the two clusters also differ in the amount of nutritional intake, however, the differences were not significant. The daily average intake of macronutrients was higher in Cluster II compared to Cluster I for protein (1.56 ± 0.296 g/kg/day vs. 1.24 ± 0.296 g/kg/day), carbohydrate (2.87 ± 0.643 g/kg/day vs. 2.09 ± 0.643 g/kg/day) and total fat (1.18 ± 0.316 g/kg/day vs. 0.77 ± 0.23 g/kg/day). Thus, the total energy intake was higher in Cluster II (28.65 ± 4.824 kcal/body weight kg/day vs. 20.87 ± 3.526 kcal/body weight kg/day), while the fiber intake was similar in the two studied groups (0.24 ± 0.005 g/kg/day and 0.21 ± 0.004 g/kg/day). The observed differences between the clusters may highlight the need for a specific nutritional recommendation for players according to their specific playing positions.

We compared the daily average nutrition intake of the athletes before and after the 4-month intervention period. However, we could not detect significant differences, probably due to the relatively high standard deviation ​​within the clusters (100–115% of the mean). The daily average intake of protein and carbohydrates showed a slight increase by 1.81 g/kg/day and 1.04 g/kg/day, respectively, while the increase of fat intake was relatively higher by 5.44 g/kg/day. The fiber intake slightly decreased after the intervention period by 0.57 g/kg/day. Overall, the daily energy intake was higher in December on average by 68.8 kcal/bodyweight kg/day.

We observed significant differences in numerous serum markers examined in the post-intervention period. We detected a significantly elevated value of hematocrit and RBC count, which would signal the increased oxygen-carrying capacity of the blood. In addition, we measured significantly increased levels of triglyceride, uric acid, and potassium, significantly decreased level of creatinine kinase and slightly decreased levels of urine and vitamin D. (Table 3). However, it is important to note that the values of the laboratory parameters were within the normal ranges at both sampling times, therefore no significant differences were detected.

**Table 3:** Changes in laboratory parameters before and after the intervention period

Variables	Before the start of the championship	After the championship	T-test value
*Laboratory parameters*			
RBC sink (mm/h)	5.158 ± 1.214	5.895 ± 1.560	1.625
Haematocrit (l/l)	0.423 ± 0.025	0.454 ± 0.024	3.970**
RBC number (T/l)	4.892 ± 0.235	5.213 ± 0.291	3.739**
Glucose (mmol/l)	4.934 ± 3.071	4.784 ± 3.057	− 0.150
Creatinine (μmol/l)	124.105 ± 71.294	109.948 ± 7.322	− 0.861
Gamma GT (U/l)	20.263 ± 8.451	19.105 ± 7.415	− 0.449
GPT U/l	30.737 ± 15.307	27.263 ± 8.918	− 0.855
GOT U/l	41.316 ± 33.721	30.895 ± 10.225	− 1.289
Ferritin (μmol/l)	23.542 ± 5.798	24.311 ± 5.711	0.412
Leukocyte (g/l)	5.488 ± 1.119	5.679 ± 1.163	0.516
Urea (mmol/l)	7.533 ± 1.530	6.521 ± 1.618	− 1.979
Creatine kinase U/l	453.421 ± 196.992	321.421 ± 156.568	− 2.287*
Uric acid (μmol/l)	280.210 ± 73.367	320.842 ± 47.754	2.023*
Hemoglobin (g/l)	168.579 ± 85.381	148.000 ± 6.807	− 1.047
Cholesterol (mmol/l)	4.186 ± 0.723	4.492 ± 0.884	1.167
HDL-cholesterol (mmol/l)	1.531 ± 0.225	1.637 ± 0.195	1.556
LDL-cholesterol (mmol/l)	2.448 ± 0.583	2.649 ± 0.764	0.909
Triglyceride (mmol/l)	0.501 ± 0.174	0.684 ± 0.152	3.458**
Sodium (mmol/l)	140.579 ± 2.269	140.000 ± 1.732	− 0.884
Potassium (mmol/l)	4.121 ± 0.303	4.332 ± 0.328	2.054*
Magnesium (mmol/l)	0.876 ± 0.069	0.867 ± 0.053	− 0.447
Calcium (mmol/l)	2.363 ± 0.107	2.356 ± 0.077	− 0.245
Vitamin D (nmol/l)	132.963 ± 40.790	111.363 ± 36.185	− 1.727
Vitamin B12 (pmol/l)	378.158 ± 183.881	381.368 ± 123.812	0.063

*RBC* red blood cell, *Gamma GT* gamma-glutamyl transferase, *GPT* glutamate-pyruvate-transaminase, *GOT* glutamate–oxaloacetate-transaminase

Statistical significance was designated by **p ≤ 0.01, *p ≤ 0.05

We examined the correlation between laboratory parameters and body composition characteristics; however, we did not find any significant correlations, probably due to the high standard deviations of the laboratory parameters. We studied the correlations between the changes in laboratory parameters before and after the intervention period and the initial anthropometric and body composition parameters. Examining the whole team (n = 19), we observed a significantly positive correlation between InBody score and changes in the amount of leukocyte cells, calcium, and vitamin B12 levels (Additional file 1: Table S2). Analyzing the clusters separately, we also detected correlation between InBody score and changes in calcium and vitamin B12 levels in cluster II (n = 8) but not in cluster I (n = 11). In general, the trends of relationships between body composition data and changes in laboratory parameters were different between the two clusters. In cluster II, the changes in glucose concentrations positively correlated with numerous body composition parameters, such as skeletal muscle mass, total body water, intracellular-, and intercellular body water ratio, basal metabolic, and minerals. At the same time, the magnesium level negatively correlated with several above listed parameters, however, these correlations could not be observed in cluster I. In addition, in cluster II the change in creatinine level showed strong positive correlation with the initial value of minerals, and Inbody score. In cluster I, the negative correlation between the changes in hemoglobin level and almost every anthropometrical and body composition parameter was remarkable; however, it is important to note, that by the creatinine and hemoglobin values, we measured a large standard deviation by the initial sampling time. Therefore, the changes of these parameters are less reliable, and further analyses are needed to reveal the correlations between these laboratory and body composition parameters (Table 4).

**Table 4:**
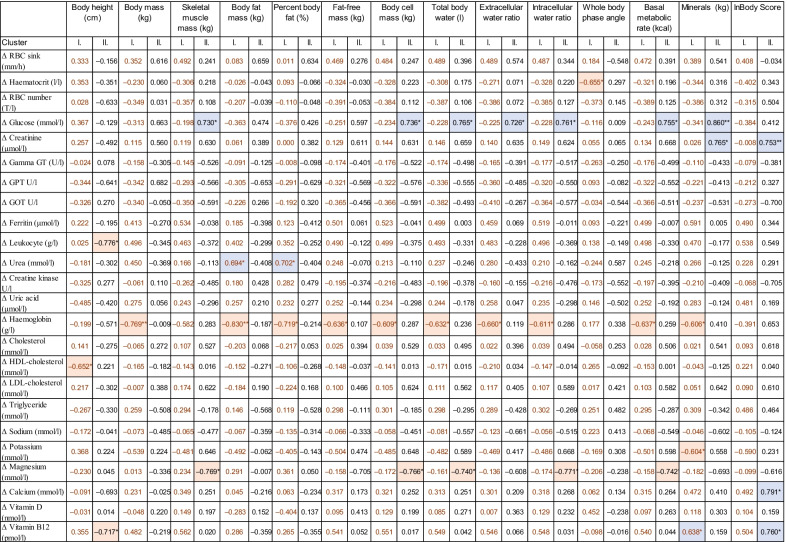
Correlation between the initial values of anthropometric and body composition characteristics and changes in laboratory parameters before and after the intervention period

For the statistical relationships Pearson correlation test was used.

*RBC* red blood cell, *Gamma GT* gamma-glutamyl transferase, *GPT* glutamate-pyruvate-transaminase, *GOT* glutamate-oxaloacetate-transaminase.

Statistical significance was designated by **p ≤ 0.01 and *p ≤ 0.05.

## Discussion

In the present work, we analyzed the anthropometric and body composition characteristics, nutrition habits, and laboratory parameters of athletes playing on the most successful Hungarian water polo team. The study was performed at the beginning of the championships and after the regular season. Each athlete had long training experience with international successes, as World championship, European Super Cup, Champions' League, and Olympic games. No scientific research has been published previously about the elite male FTC water polo team. Analysis on self-reported health status of the participants showed significant differences in numerous questions in the post-intervention period. Overall, their perceived health status and physical condition had changed in an unfavorable way. The explanation to this may be in connection with the increase in the number of matches during the competitive season, which requires high consistent athletic performance, and moreover, added mentally demanding technical and tactical elements to their everyday training.

We observed strong positive correlations between the anthropometric and body composition characteristics. The correlation between the body composition characteristics and the physical performance of handball players and swimmers had been studied [16, 17]. These studies found that body composition characteristics were associated with performance; therefore, these results can help the work of coaches to achieve more optimal sports performance for handball players and swimmers.

Due to the specific characteristics of water polo, results of other sports could not be directly applied. Thus, our results can be directly compared only with the results of other studies performed on water polo players. However, to the best of our knowledge, only a few studies have been published in this field. In a former study, Kondrič et al. [7] examined the anthropometric and body composition characteristics, physical condition and fitness of 110 junior water polo players with different playing positions. Based on their results, centers had significantly higher body weight, BMI, and larger subscapular skinfold compared to other players. In that study, the players’ physical condition was measured by swimming tests. They found that the shooters achieved the best test results in 25 m and 400 m sprints, but without significant differences [7]. However, swimming tests are not suitable to describe the performance status of players during the training sessions and games, which both consist of technical and tactical components and thus require further skills. The swimming tests can characterize only the physical status of players. Unfortunately, the accurate monitoring of players during training and games is still unresolved, making it difficult to collect performance data.

In our analysis, using cluster analysis, we determined two significantly different clusters based on players’ anthropometric and body composition characteristics. Cluster I included goalkeepers and wingers, while Cluster II consisted of centers, shooters, and defenders. Cluster I players were 5 cm shorter on average, while their mean body weight, skeletal muscle mass and body fat mass data were lower by 19 kg, 7 kg and 7 kg, respectively, compared to height, body weight, skeletal muscle mass and body fat mass of players classified into cluster II. These results are consistent with the results described by Kondrič et al. and can be explained by the fact that the playing position of goalkeepers and wingers requires more dynamism, explosiveness and agility. In former studies, specific anthropometric differences of athletes in different playing positions have also been described for adult male elite water polo players. These studies have also confirmed that athletes playing in center or defender positions are taller and have the highest BMI within the team [18, 19]. These examples highlight significant anthropometric differences between playing positions and can give support to the development of position-specific training programs and thus, nutritional recommendations. In addition to the abovementioned results, we found a significant decrease in the mineral composition of athletes after the 4-month intervention period, which parameter represents the osseous and non-osseous mineral amounts. The decrease in the amount of minerals can be explained by the parallel decrease in vitamin D level, which has an impact on bone mass acquisition. On the other hand, the adequate protein intake is also assumed to support bone metabolism [20], which was lower than the recommended amount for athletes.

As performance might be strongly influenced by nutrition intake, the athletes’ dietary habits were also analyzed. Although we detected a slight differences in macronutrient consumption between the two clusters, they were not significant. However, we would like to suggest that nutrition recommendations should reflect the different physical- and, therefore, nutritional demands of Cluster I and II. We calculated that the athletes in Cluster II had higher daily protein, carbohydrate, and fat intake, which then resulted in higher overall calorie intake. Due to the high demands of elite sports, such as the high number of daily in-water and/or dryland conditioning trainings for water polo players, the level of blood sugar decreases during and/or after the exercises. Therefore, adequate carbohydrate intake can reduce the incidence of exercise-induced hypoglycemia. Muscle glycogen depletion depends on the duration and intensity of exercise, suggesting that the recommended daily carbohydrate intake for elite male athletes is 8–12 g/kg/day for optimal replenishment of glycogen stores [1, 2, 4]. Based on our results, rather alarmingly, athletes did not get close to this recommended carbohydrate intake, which was 2.09 g/kg/day in Cluster I and 2.87 g/kg/day in Cluster II. The daily protein intake also did not meet the recommended rate of 1.4–2.0 g/kg/day consumption in Cluster I, which means an average of 1.24 kcal/kg/day [1]. According to the recommendations, athletes need to consume almost twice as much protein daily as the sedentary population to maintain appropriate protein synthesis and energy production. On the other hand, the required amount is given separately for athletes doing endurance or strength sport, as the intensity and length of training also influence the needs. In addition, optimal timing of protein intake has also an important role. It should be consumed before and after the training, and regularly 3–5 times during the day. Protein intake is especially noteworthy because the body is unable to store amino acids such as fatty acids or carbohydrates [20]. The recommended fat intake is a maximum of 30% of the total daily calorie intake regardless of training intensity [21], which was appropriate in both clusters. The players’ fiber intake was also within the recommended range. In summary, lower carbohydrate and protein consumption was reported, particularly by athletes in Cluster I which suggests position-specific and/or personalized nutritional plans. However, in elite sports, it is regular for players to eat the same meal after training and matches, without paying attention to individual nutritional needs. A former study performed among elite woman volleyball players also showed that the daily average energy intake of athletes, including especially the carbohydrate consumption, was lower than the recommended amount [22]. Therefore, it would be important for the players to receive personalized nutritional recommendations by experts. However, it is important to emphasize that these athletes achieved numerous international victories regardless the above-mentioned nutritional results. Therefore, the international nutritional recommendations should be treated with caution. In the literature, no position-specific nutritional recommendation for water polo players has been described, however the differences in anthropometric and body composition parameters highlighted the need for more specific recommendations. In the present study, the number of athletes stratified by playing positions is limited, therefore, we would like to extend the analysis to more water polo teams from Hungary. In the future, we would like to focus much more on the individual nutritional requirements of athletes according to their playing positions, considering the actual training length and intensity.

Nutritional deficiencies can be identified by monitoring laboratory parameters. During the 4-month research period, the strength endurance training was replaced with more tactical training and games. In line with this, after the end of the regular season, we detected an elevated level of triglyceride, uric acid, and potassium, and a decreased level of creatinine kinase. In addition, we measured a slightly decreased levels of urea and Vitamin D (*p* < 0.1). A higher triglyceride level might be caused by the relatively higher fat intake (5.44 g/kg/day), while significantly higher uric acid level by the higher training volume and exercise intensity [23]. The high potassium concentration in the blood is a response to muscle contraction and therefore also associated with higher training volume [24]. The insufficient protein intake can also be determined with the low urea level, while the decreased level of vitamin D may be associated with the overload of players. Vitamin D has an effect on muscle activity, a deficiency which can cause weakness and influence recovery time after exercise. Therefore, relevant vitamin intake is needed to achieve the optimal athletic performance [12, 25]. We examined the correlation between the initial anthropometric and body composition parameters and changes in laboratory parameters before and after the championship. We detected different pattern in the changes in glucose and magnesium between the two clusters. Higher glucose level may be occurred by higher carbohydrate consumption in cluster II. These results may confirm the differences between the two clusters, however further analyses are needed to reveal the factors caused the differences, which was observed mostly in cluster II.

## Conclusions

In the present study, we examined the anthropometric and body composition characteristic, dietary habits, and laboratory parameters of elite male water polo players competing internationally on the most successful Hungarian team. Based on their body composition, the examined athletes could be classified into two distinct clusters. The clusters related to the playing positions strongly, and they showed differences in their dietary habits. Although these players achieved international successes, their daily protein and carbohydrate intake was much lower than the international recommendations. Due to the small number of participants, our findings are limited, and need to be validated. Regardless, the study can be an important basis for establishing body compositional and nutritional recommendations for players with distinct playing positions to improve their performance.

## Supplementary Information

**Additional file 1: Table S1.** SF-36 Questionnaire according to Ware et al. Statistical significance was designated by ***p≤0.01, **p≤ 0.05, *p≤ 0.1. **Table S2.** Correlation between the initial values of anthropometric and body composition characteristics and changes in laboratory parameters before and after the intervention period. For the statistical relationships Pearson correlation test was used. *RBC* red blood cell, *Gamma GT* gamma-glutamyl transferase, *GPT* glutamate-pyruvate-transaminase, *GOT* glutamate-oxaloacetate-transaminase. Statistical significance was designated by *p≤ 0.05.

## Data Availability

All data generated and analyzed during the current study are included in this published article [and its Additional file]. The raw datasets are available from the corresponding author on reasonable request. (Dr. Péter Fritz; efkfritz@uni-miskolc.hu).
