# Supplementary material for: Hungarian male water polo players’ body composition can predict specific playing positions and highlight different nutritional needs for optimal sports performance
Source: BMC Sports Sci Med Rehabil. 2022 Sep 5;14:165. doi: 10.1186/s13102-022-00560-9 (PMC9447334; doi:10.1186/s13102-022-00560-9)
Supplement: Supplementary file 1 — Additional file 1: Table S1. SF-36 Questionnaire according to Ware et al. Statistical significance was designated by ***p≤0.01, **p≤ 0.05, *p≤ 0.1. Table S2. Correlation between the initial values of anthropometric and body composition characteristics and changes in laboratory parameters before and after the intervention period. For the statistical relationships Pearson correlation test was used. RBC red blood cell, Gamma GT gamma-glutamyl transferase, GPT glutamate-pyruvate-transaminase, GOT glutamate-oxaloacetate-transaminase. Statistical significance was designated by *p≤ 0.05. [file 13102_2022_560_MOESM1_ESM.docx]

**Additional file**

**Supplementary Table 1.** SF-36 Questionnaire according to Ware et al. Statistical significance was designated by *** p≤0.01, ** p≤ 0.05, * p≤ 0.1.

| Concepts | Aug  (mean) | Dec  (mean) | F-value | P-value | Mann-Whitney test (Z-value) | P-value |
| --- | --- | --- | --- | --- | --- | --- |
| 1. In general, would you say your health is: | 4.15 | 2.42 | 29.34 | <0.001 | 3.821 | *** |
| 1. Compared to one year ago, how would you rate your health in general now? | 3.57 | 2.52 | 13.85 | <0.001 | 3.423 | *** |
| 1. The following items are about activities you might do during a typical day. Does your health now limit you in these activities? I | | | | | | |
| - 1. Vigorous activities, such as running, lifting heavy objects, participating in strenuous sport | 1.21 | 2.36 | 29.43 | <0.001 | -4.025 | *** |
| - 1. Moderate activities, such as moving a table, pushing a vacuum cleaner, bowling etc. | 1 | 2.42 | 47.2 | <0.001 | -4.613 | *** |
| - 1. Lifting or carrying groceries | 1 | 2.42 | 47.2 | <0.001 | -4.132 | *** |
| - 1. Climbing several flights of stairs | 1.1 | 2.31 | 31.53 | <0.001 | -4.646 | *** |
| 3.5 Climbing one flight of stairs | 1 | 2.47 | 50.4 | <0.001 | -4.564 | *** |
| - 1. Bending, kneeling, or stooping | 1 | 2.31 | 41.98 | <0.001 | -4.416 | *** |
| - 1. Walking more than 1 km | 1.05 | 2.42 | 41.11 | <0.001 | -4.613 | *** |
| - 1. Walking more than 100 m? | 1 | 2.42 | 47.2 | <0.001 | -4.613 | *** |
| - 1. Walking 100 m? | 1 | 2.42 | 47.2 | <0.001 | -4.613 | *** |
| - 1. Bathing or dressing yourself | 1 | 2.42 | 47.2 | <0.001 | -4.613 | *** |
| 1. During the past 4 weeks, have you had any of the following problems with your work or other regular daily activities as a result of your physical health? | | | | | | |
| - 1. Cut down the amount of time you spent on work or other activities | 1.26 | 2.57 | 32.33 | <0.001 | -4.116 | *** |
| - 1. Accomplished less than you would like | 1.24 | 2.47 | 34.33 | <0.001 | -4.178 | *** |
| - 1. Were limited in the kind of work or other activities | 1.1 | 1.42 | 36.06 | <0.001 | -4.237 | *** |
| - 1. Had difficulty performing the work or other activities | 1.1 | 1.47 | 38.75 | <0.001 | -4.297 | *** |
| 1. During the past 4 weeks, have you had any of the following problems with your work or other regular daily activities as a result of any emotional problems (such as feeling depressed or anxious)? | | | | | | |
| - 1. Cut down the amount of time you spent on work or other activities | 1.05 | 2.47 | 44.03 | <0.001 | -4.463 | *** |
| - 1. Accomplished less than you would like | 1.1 | 2.36 | 33.66 | <0.001 | -4.182 | *** |
| - 1. Didn't do work or other activities as carefully as usual | 1.05 | 2.41 | 44.56 | <0.001 | -4.560 | *** |
| 1. During the past 4 weeks, to what extent has your physical health or emotional problems interfered with your normal social activities with family, friends, neighbors, or groups? | 1.42 | 1.67 | 5.26 | <0.001 | 2.010 | ** |
| 1. How much bodily pain have you had during the past 4 weeks? | 2.26 | 1.89 | 1.39 | 0.123 | 0.954 |  |
| 1. During the past 4 weeks, how much did pain interfere with your normal work (including both work outside the home and housework)? | 1.47 | 1.89 | 3.27 | <0.001 | 1.724 | ** |
| 1. These questions are about how you feel and how things have been with you during the past 4 weeks. For each question, please give the one answer that comes closest to the way you have been feeling. How much of the time during the past 4 weeks | | | | | | |
| - 1. Did you feel full of pep? | 3.75 | 4.42 | 5.7 | <0.001 | -1.251 | ** |
| - 1. Have you been a very nervous person? | 1.94 | 2.78 | 2.14 | 0.012 | 1.922 | ** |
| - 1. Have you felt so down in the dumps that nothing could cheer you up? | 1.42 | 1.52 | 0.12 | 0.89 | 0.378 |  |
| - 1. Have you felt calm and peaceful? | 3.31 | 4.15 | 3.25 | <0.001 | -1.545 | ** |
| - 1. Did you have a lot of energy? | 3.36 | 4.15 | 3.88 | <0.001 | -1.872 | ** |
| - 1. Have you felt downhearted and blue? | 1.78 | 2.63 | 2.65 | <0.001 | 1.820 | ** |
| - 1. Did you feel worn out? | 3.68 | 4.54 | 4.05 | <0.001 | 1.328 | ** |
| - 1. Have you been a happy person? | 3.42 | 3.05 | 0.74 | 0.98 | -0.956 |  |
| - 1. Did you feel tired? | 2.05 | 3.73 | 12.34 | <0.001 | 0.901 | ** |
| 1. During the past 4 weeks, how much of the time has your physical health or emotional problems interfered with your social activities (like visiting with friends, relatives, etc.)? | 1.57 | 1.89 | 0.8 | 0.75 | -2.964 | *** |
| - 1. I seem to get sick a little easier than other people. | 4.38 | 4.68 | 0.93 | 0.78 | -1.279 | ** |
| - 1. I am as healthy as anybody I know. | 1.63 | 1.57 | 0.03 | 0.98 | -0.930 | ** |
| - 1. I expect my health to get worse. | 3.14 | 4.5 | 2.88 | 0.00 | 0.278 |  |
| - 1. My health is excellent. | 3.14 | 4.50 | 2.88 | 0.00 | -0.404 |  |

SF-36 Response Choices

1. Excellent, Very Good, Good, Fair, Poor

2. Much better now than one year ago, Somewhat better now than one

year ago, About the same as one year ago, Somewhat worse now than

one year ago, Much worse than one year ago

3. Yes, Limited a lot; Yes, Limited a little; No, Not limited at all

4.1-4. Yes, No

5.1-3. Yes, No

6. Not at all, Slightly, Moderately, Quite a bit, Extremely

7. None, Very mild, Mild, Moderate, Severe, Very severe

8. Not at all, A little bit, Moderately, Quite a bit, Extremely

9. All of the time, Most of the time, A good bit of the time, Some of the

time, A little of the time, None of the time

10. All of the time, Most of the time, Some of the time, A little of the time,

None of the time

1. Definitely true, Mostly true, Don't know, Mostly false, Definitely

**Supplementary Table 2.** Correlation between the initial values of anthropometric and body composition characteristics and changes in laboratory parameters before and after the intervention period. For the statistical relationships Pearson correlation test was used. RBC – red blood cell. Gamma GT – gamma-glutamyl transferase, GPT – glutamate-pyruvate-transaminase, GOT – glutamate-oxaloacetate-transaminase. Statistical significance was designated by * p≤ 0.05.

**Reference**

Ware JE Jr, Gandek B. Overview of the SF-36 Health Survey and the International Quality of Life Assessment (IQOLA) Project. J Clin Epidemiol. 1998 Nov;51(11):903-12. doi: 10.1016/s0895-4356(98)00081-x.
